# Misleading Rare Case of Idiopathic Hypertrophic Pachymeningitis

**DOI:** 10.1155/2024/5561686

**Published:** 2024-03-13

**Authors:** Ahmad Rezaee Azandaryani, Amir Mohammad Salehi

**Affiliations:** ^1^Department of Radiology, School of Medicine, Hamadan University of Medical Sciences, Hamadan, Iran; ^2^Student Research Committee, Hamadan University of Medical Sciences, School of Medicine, Hamadan, Iran

## Abstract

Idiopathic hypertrophic pachymeningitis (IHP) is a rare disease with diffuse thickening of the dura mater that has no specific clinical symptoms and manifestations and it causes neurosurgeons to misdiagnose. A 4-year-old girl presented at the emergency room of our hospital with speech difficulty and severe headache. Head computed tomography scans (CT scan) on admission revealed a large fluid collection over the right temporoparietal region with mass effect, and the neurosurgeon drained it with the initial diagnosis of subdural hematoma. However, the postoperative CT scan demonstrated the failure of surgical drainage; therefore, magnetic resonance imaging (MRI) was requested for the patient. MRI identified diffuse nodular dural thickening with internal septations and different internal hemorrhagic stages on the right side with no evidence of brain parenchymal involvement and according to the serology and autoimmune screening tests, and IHP was diagnosed for the patient. The patient underwent craniotomy. There was an immediate improvement of neurologic symptoms. The patient had good clinical and radiologic outcome at 3 -months follow-up. IHP should be part of the differential diagnosis of some cases of noncommunicating hydrocephalus; however, the rarity of the disease and the absence of specific clinical symptoms make the diagnosis difficult.

## 1. Introduction

Hypertrophic pachymeningitis (HP) is a neurological disease characterized by focal or diffuse thickening of the dura mater, with or without inflammation. Primary or idiopathic hypertrophic pachymeningitis (IHP) does not have an identifiable cause. Secondary HP may follow a known cause such as trauma, infection (neurosyphilis, tuberculosis, fungal infections, syphilis, and Lyme's disease), tumors (dural carcinomatosis, meningiomaen plaque, and lymphoma), autoimmune diseases (Sjogren's and IgG4-related diseases and collagen vascular disorders), and miscellaneous disorders such as sarcoidosis, mucopolysaccharidosis, intracranial hypotension syndrome, and intrathecal drug administration [1, 2].

IHP is an extremely rare disorder with nonspecific clinical symptoms and manifestations, making diagnosis difficult [3]. Herein, we present a case of IHP in a 4-year-old girl which caused the initially misdiagnosed as subdural hematoma.

## 2. Case Presentation

Our case report describes a 4-year-old girl who presented at the emergency room of Be'sat Hospital with left-side hemiplegia and seizure. The patient had a history of hydrocephalus at birth and had a shunt implanted. The patient was conscious during the physical examination. Vital signs included a body temperature of 37°C, a heart rate of 112 beats per minute, a respiratory rate of 18 beats per minute, and a blood pressure of 110/70 mmHg.

She had a full neurological examination, which reveals aphasia and facial and left upper limb paresthesia. Her systemic examination was normal and in particular, there was no evidence of lymphadenopathy, organomegaly, or cutaneous features of connective tissue disease. Lumbar puncture showed increased intracranial pressure.

**Figure 1:**
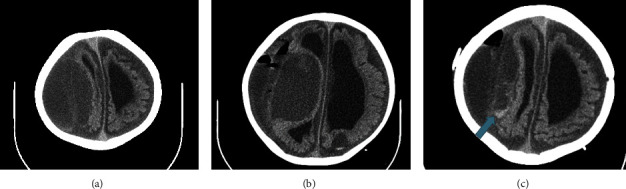
Nonenhanced axial CT scan demonstrates large right temporoparietal fluid collection (a). Nonenhanced axial postop CT scan demonstrates failure of surgical drainage due to the presence of dural layer in deep portion of the collection (b). Follow-up nonenhanced axial CT scan showed interval hemorrhage (blue arrow) in the collection (c).

**Figure 2:**
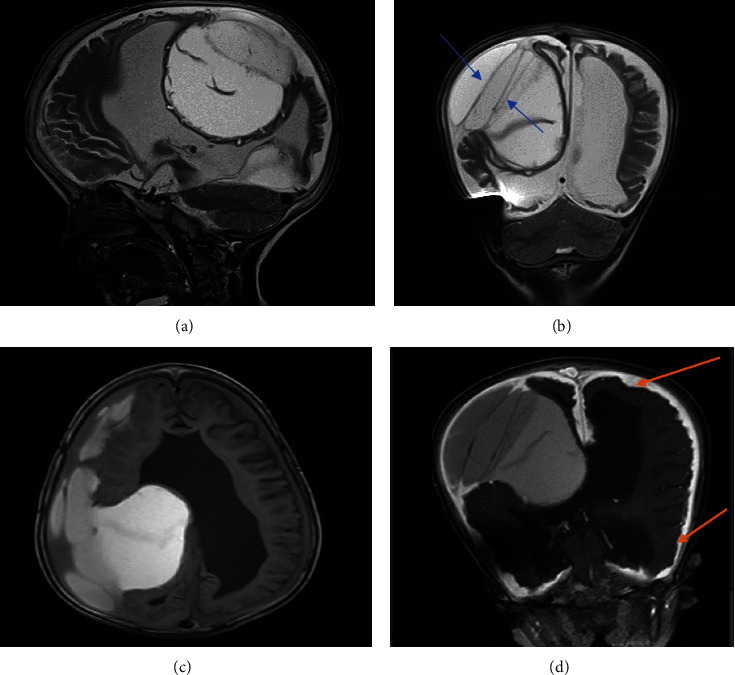
Sagittal T2W (a) and coronal T2W images demonstrated right pariental complex fluid collection with internal septation (blue arrow) and different internal hemorrhagic stages and mass effect (b). Axial FLAIR image demonstrated that collection over right temporoparietal extraaxial collection with mass effect has focal internal layering (c). Contrast-enhanced T1W image showed diffuse nodular dural thickening and dural enhancement (orange arrow) (d).

A head computed tomography scan (CT scan) was quickly requested for the patient, in which a large fluid collection over the right temporoparietal region with mass effect was observed, and the neurosurgeon drained it with the initial diagnosis of subdural hematoma (Figure 1(a)). However, the postoperative CT scan demonstrated the failure of surgical drainage due to the presence of a dural layer in the deep portion of the collection (Figure 1(b)). After 3 days, a follow-up CT scan showed an interval hemorrhage in the collection (Figure 1(c)). Therefore, magnetic resonance imaging (MRI) was requested for the patient. MRI identified diffuse nodular dural thickening with internal septations and different internal hemorrhagic stages (hypertrophic pachymeningitis) on the right side (Figures 2(a)–2(d)) with no evidence of brain parenchymal involvement.

Following consultation with the infectious diseases and rheumatology team, serology/PCR for varicella zoster virus, *Mycoplasma pneumonia*, Haemophilus influenza type B, human herpesvirus 6 (PCR), enterovirus (PCR), *Borrelia burgdorferi*, cytomegalovirus (PCR), HIV, syphilis, and Epstein–Barr virus, and autoimmune screening test (ANA, anti-dsDNA, anti-SSA, anti-SSB, antiphospholipid, anticardiolipin, and ANCA) were requested, which did not indicate an infectious or immune cause for the hypertrophic pachymeningitis; therefore, IHP was diagnosed for the patient.

Due to noncommunicating hydrocephalus, the patient was taken back to the operating room and underwent craniotomy surgery. A dural biopsy through a craniectomy was performed, which confirmed the diagnosis of IHP. The patient experienced prompt clinical and neurologic improvement with the resolution of the preoperative symptoms related to increased intracranial pressure. The patient was discharged home on 60 mg of prednisolone orally daily.

She was reviewed in the outpatient clinic a month after discharge and was doing well. Her headaches and paresthesia had resolved completely. At 3 months from symptom presentation, while on a slow steroid taper (10 mg daily, 6 months weaning protocol), repeat imaging continues to demonstrate no active dural disease.

## 3. Discussion

IHP is an extremely rare disorder that predominantly affects male patients and is characterized by inflammatory fibrosis and localized or diffuse dura mater thickening without underlying disease [4, 5]. In patients with IHP, headache, nausea, and vomiting are the most common symptoms. Headache is mainly due to inflammation of the dura mater but can also be due to raised intracranial pressure. Visual loss (due to optic neuropathy subsequent optic atrophy and the third, fourth, and sixth cranial nerves involvement), seizures, encephalopathy, and hemiparesis are other symptoms reported in IHP patients [6].

IHP mimicked other common neurologic conditions such as prolactinoma with recurrent vision loss, neurosarcoidosis, atypical lymphoplasmacytic-rich meningioma, lymphoplasmacytic-rich meningioma, subdural hematoma, and subacute subtentorial hematoma [5]. IHP misdiagnosed as subdural hematoma is not uncommon; however, IHP presenting as subdural hematoma is unusual [7]. In our case, the initial CT scan showed features of a subdural hematoma, leading the surgeon to make a misdiagnosis.

The definitive diagnosis is based on MRI or brain biopsy of the thickened dura mater. Biopsy reveals interstitial fibrosis and inflammatory cell infiltration. Enhanced MRI is the most valuable test in the diagnosis of IHP. When the dura is not obviously thick, IHP may not be diagnosed on a CT scan and/or MRI without contrast [8]. The lesions appeared hypointense or isointense on T1-weighted sequences, hypointense on T2-weighted sequences, hypointense on FLAIR sequences, and hypointense on DWI [9]. Postcontrast enhancement is usually seen. Enhanced MRI can be used to evaluate the effect of therapy [10].

Although no specific protocol has been proposed for the treatment of IHP so far, however, by reviewing the available literature, two conservative treatment lines have been defined based on the disease mechanism, symptoms, and patient conditions. The first line of treatment includes the administration of steroids, prednisolone, with an initial dose of 42.7 mg/day and a maintenance dose of 12.4 mg/day for long-term treatment. In cases resistance to steroids or recurrence when steroids are tapered, an immunosuppressant, such as cyclophosphamide or methotrexate, is a second choice to treat steroid-refractory IHP [5]. However, sometimes, conservative treatment is not effective enough to prevent severe complications in life-threatening conditions, such as noncommunicating hydrocephalus, prompt surgical intervention may be necessary. In our case, subtotal resection surgery was performed and then treatment with prednisolone was administered after pathologic diagnosis [11].

So far, including our case, this is the third reported case of IHP with hydrocephalus (Table 1) [12, 13]. 2 cases were children and one case was an adult. In the reported cases, headache is the most common symptom of this disease. In the patient reported by Aburahma et al. [12], unlike our patient, the patient was treated with conservative treatment and without craniectomy. Also, our patient had good clinical and radiologic outcome at the 3 -months follow-up.

## 4. Conclusion

IHP should be a part of the differential diagnosis of some cases of noncommunicating hydrocephalus; however, the rarity of the disease and the absence of specific clinical symptoms make the diagnosis difficult.

## Figures and Tables

**Table 1:** Summary of case reports on noncommunicating hydrocephalus with IHP.

First author, year (references)	Age (years)/Sex	Symptoms/Signs	Treatment	Outcome	Postoperative treatment
Aburahma et al., 2009 [12]	3.5/Male	Irritability, vomiting, neck stiffness, headache, visual decline	Corticosteroid, cyclophosphamide, ventriculoperitoneal shunt	Poor at 1-year follow-up	Intrathecal cytarabine
Huang et al. 2017 [13]	60/Female	Headache, multiple cranial nerve palsies, gait instability, dizziness	Suboccipital craniectomy, C1 laminectomy	Gradual improvement at 1-month follow-up; good at 1-year follow-up	Corticosteroid
Our case	4/Female	Headache, aphasia, facial and eft upper limb paresthesia	Craniectomy	Gradual improvement at 1-month follow-up; very good at 3-month follow-up	Corticosteroid

## Data Availability

Access to data is permitted with the author's permission.

## Abbreviations

IHP: Idiopathic hypertrophic pachymeningitis
MRI: Magnetic resonance imaging
CT: Computerized tomography.
